# E‐waste: mechanisms of toxicity and safety testing

**DOI:** 10.1002/2211-5463.13863

**Published:** 2024-07-10

**Authors:** Chukwuebuka Eze, Mathieu Vinken

**Affiliations:** ^1^ Entity of In Vitro Toxicology and Dermato‐Cosmetology, Department of Pharmaceutical and Pharmacological Sciences, Faculty of Medicine and Pharmacy Vrije Universiteit Brussel Brussels Belgium

**Keywords:** E‐waste, human health, new approach methodologies, risk assessment, toxicity pathways

## Abstract

Currently, information on the toxicity profile of the majority of the identified e‐waste chemicals, while extensive and growing, is admittedly fragmentary, particularly at the cellular and molecular levels. Furthermore, the toxicity of the chemical mixtures likely to be encountered by humans during and after informal e‐waste recycling, as well as their underlying mechanisms of action, is largely unknown. This review paper summarizes state‐of‐the‐art knowledge of the potential underlying toxicity mechanisms associated with e‐waste exposures, with a focus on toxic responses connected to specific organs, organ systems, and overall effects on the organism. To overcome the complexities associated with assessing the possible adverse outcomes from exposure to chemicals, a growing number of new approach methodologies have emerged in recent years, with the long‐term objective of providing a human‐based and animal‐free system that is scientifically superior to animal testing, more effective, and acceptable. This encompasses a variety of techniques, typically regarded as alternative approaches for determining chemical‐induced toxicities and holds greater promise for a better understanding of key events in the metabolic pathways that mediate known adverse health outcomes in e‐waste exposure scenarios. This is crucial to establishing accurate scientific knowledge on mixed e‐waste chemical exposures in shorter time frames and with greater efficacy, as well as supporting the need for safe management of hazardous chemicals. The present review paper discusses important gaps in knowledge and shows promising directions for mechanistically anchored effect‐based monitoring strategies that will contribute to the advancement of the methods currently used in characterizing and monitoring e‐waste‐impacted ecosystems.

## Graphical abstract

This review examines the present knowledge of the likely underlying toxicity mechanisms associated with exposure to e‐waste, with a focus on toxic effects on organs, organ systems, and overall organism impacts. In addition, important gaps in knowledge and intriguing opportunities for safety testing approaches that would allow for accurate monitoring and assessment of e‐waste exposure in compromised ecosystems were explored.

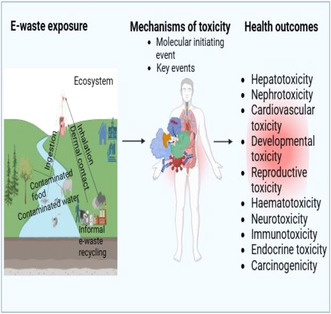

**Abbreviations**
AOP: adverse outcome pathway
BPA: bisphenol A
DNA: deoxyribonucleic acid
DP: dechlorane plus
DTs: digital twins
ERK: extracellular‐signal‐regulated kinase
GSH: reduced glutathione
IQ: intelligent quotient
JNK: *c*‐jun N‐terminal kinase
KEs: key events
KIM‐1: kidney injury molecule‐1
MAPK: mitogen‐activated protein kinase
MIEs: molecular initiating events
NAG: *N*‐acetyl‐β‐d‐glucosaminidase
NAMs: new approach methodologies
NFRs: novel flame retardants
NGAL: neutrophil gelatinase‐associated lipocalin
PAHs: polycyclic aromatic hydrocarbons
PBDEs: polybrominated diphenyl ethers
PCBs: polychlorinated biphenyls
PCDD/Fs: polychlorinated dibenzo‐(1,4)‐dioxins, and dibenzofurans
T. Bil: total bilirubin
TBEP: tris(2‐butoxyethyl) phosphate
TCEP: tris(2‐chloroethyl) phosphate
TDCPP: tris(1,3‐dichloroisopropyl) phosphate
TNBP: tri‐n‐butyl phosphate
TNF‐α: tumor necrosis factor alpha
TOCP: tri‐o‐cresyl phosphate
TPP: triphenyl phosphate
TSH: thyroid‐stimulating hormone
UACR: urinary albumin/creatinine ratio
β2‐MG: Beta‐2‐microglobulin

End‐of‐life electrical and electronic equipment is typically exported from developed countries to developing regions, therefore increasing the destination countries' total burden of electronic trash (e‐waste) [1, 2, 3]. The need for recycling these outmoded products is constantly increasing, as they contain valuable elements such as copper, iron, silicon, nickel, and gold that may be recovered [3, 4]. Unfortunately, both formal and informal e‐waste recycling processes used in developed and developing countries, respectively, often lead to the discharge of varying amounts of complex combinations of harmful chemicals into e‐waste recycling sites and the surrounding environment [4, 5, 6]. The frequent technical advancements in the production of electrical and electronic equipment make the specific chemical composition of e‐waste vary regularly. Furthermore, because other business activities that could contribute to the pollution load also occur in most e‐waste recycling sites, site‐specific chemical pollution scenarios might vary in substance and amount as well, hence making detailed chemical and toxicological characterization of e‐waste‐impacted ecosystems a challenging and time‐consuming endeavor [7, 8, 9, 10].

There is an increasing corpus of direct evidence that e‐waste exposure disrupts biological processes and leads to adverse health outcomes [5, 11, 12, 13, 14, 15]. Humans and other organisms that play important roles in trophic interactions are usually exposed to the different chemicals found in e‐waste or released during crude e‐waste recycling procedures (Fig. 1). Although human exposure routes could differ depending on the e‐waste recycling method employed and type of e‐waste chemical released, the combined inhibitory, additive, or synergistic effects of multiple exposures can all have an impact on overall health outcomes [4]. Environmental toxicology is a field that is critical to the development of effective and reliable methods for assessing the health risk associated with chemical exposures. Bioanalytical capabilities are improving rapidly worldwide by employing effect‐based monitoring approaches *in vitro* and *in vivo* to determine adverse effects of chemicals at different levels of biological organization, and it usually necessitates the use of multiple endpoints to determine the various mechanisms of action of the chemicals in both single and mixture forms [16]. Interestingly, the use of advanced alternative approaches has recently emerged in *in vitro* toxicity assessment for chemical substances [17, 18] and holds greater promise for applications in different areas of toxicology as they are eligible for adjusting risk assessment inputs for combination interactions without exponentially expanding the number of animals required to determine toxicological reference values for predicting human impacts, due to their ease of use and growing standardization [17].

**Fig. 1:**
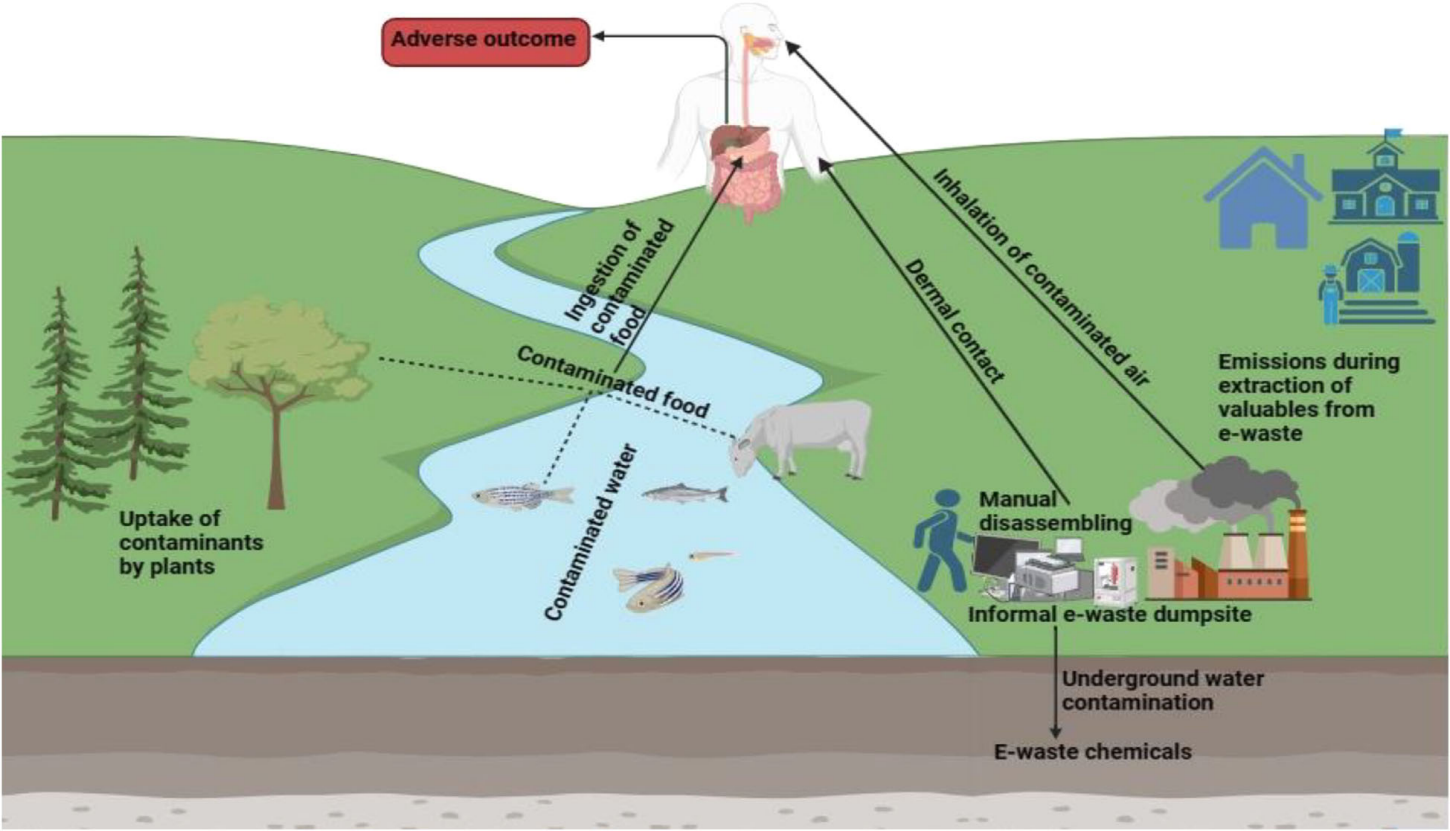
Human exposure pathways during and after informal e‐waste recycling. E‐waste chemicals are typically found in complex environmental matrices at e‐waste sites, posing significant environmental and health concerns to nearby populations and biota. It is widely acknowledged that informal e‐waste recycling and disposal in landfills pose serious threats to both the environment and human health. As such, to assess the human health risks associated with e‐waste contamination at e‐waste sites, it is critical to understand the exposure pathways. The 3 major exposure pathways include inhalation of fine and coarse particles released during the extraction of valuable materials from e‐waste, ingestion of e‐waste‐contaminated food, and dermal contact with hazardous e‐waste substances via the skin.

Human exposure to e‐waste is associated with higher levels of many toxic chemicals, such as lead, cadmium, mercury, manganese, chromium, nickel, polycyclic aromatic hydrocarbons (PAHs), polybrominated diphenyl ethers (PBDEs), polychlorinated biphenyls (PCBs), dechlorane plus (DP), polychlorinated dibenzo‐(1,4)‐dioxins, and dibenzofurans (PCDD/Fs), novel flame retardants (NFRs), bromophenols, perchlorate and thiocyanate, polybrominated biphenyls, phthalate esters, bisphenols, and organophosphates [19, 20, 21, 22, 23, 24, 25, 26, 27, 28, 29, 30, 31, 32, 33, 34, 35, 36, 37, 38, 39, 40, 41, 42, 43, 44, 45, 46, 47, 48, 49]. The accumulation of evidence of distinct and identifiable health concerns connected with e‐waste exposures has prompted an upsurge in e‐waste risk assessment studies and campaign. While scientific evidence on the negative impacts of exposure to many recognized e‐waste chemicals is numerous and expanding, it is obviously deficient, particularly at the cellular and molecular levels. More importantly, the toxicity of the complex chemical mixes that humans are expected to encounter during and after informal e‐waste recycling, as well as the underlying mechanisms of action, remain largely ununderstood.

The present review paper underlines studies that explored real‐life e‐waste chemical exposure scenarios, including those that examined the effects of exposure to e‐waste‐impacted matrices and studies that identified the mechanisms of action associated with exposure to e‐waste chemicals or e‐waste chemical mixtures, as the combinations of the studies are important for potential generalizations and could also provide predictions of harmful effects associated with e‐waste exposure that have not yet been determined. As a result, this review study highlights the current understanding of the probable underlying toxicity mechanisms associated with e‐waste exposures, with an emphasis on toxic reactions to organs, organ systems, and total organism impacts. Furthermore, significant knowledge gaps and promising possibilities for mechanistically anchored safety testing strategies that will enable adequate characterization and assessment of e‐waste exposure in affected ecosystems were examined.

## Method

A complete literature search was independently undertaken on 11 databases (Web of Science, National Library of Medicine, EMBASE, MEDLINE, PubMed, Scopus, Cochrane Library, PsycNET, Google Scholar, ScienceDirect, and Biomed Central) during January – May 2024 for articles published in English. The following keywords were used for the search: “chemical characterizations of e‐waste,” “toxicities of e‐waste chemicals,” “harmful e‐waste exposure scenarios,” “metabolic effects of e‐waste chemicals,” “toxicity mechanisms of e‐waste chemicals,” “modes of action of heavy metals and organic pollutants associated with e‐waste,” “heavy metal toxicity,” “toxicity of flame retardants,” “adverse human effects associated with e‐waste exposure,” “molecular interactions and key events associated with exposure to e‐waste chemicals,” “adverse outcome pathway (AOP) networks,” “relevance of new approach methodologies (NAMs) in chemical risk assessment,” “NAMs framework,” and “regulatory acceptance of NAMs.” Overall, the search scope was limited to published literature that focused on toxicity and toxicity mechanisms associated with harmful exposures to e‐waste chemicals. Studies that reported outcomes in humans, including *in vivo* and *in vitro* experimentation, were included. However, abstracts, editorials, correspondence, prefaces, commentary, and studies that do not report any health outcomes in relation to e‐waste chemicals were not included. Following preliminary title and abstract screening, full‐text assessment of the relevant articles was performed based on the predetermined inclusion and exclusion criteria to ascertain the suitability and relevance of the considered papers (Table 1). Screening of the papers for eligibility was undertaken according to PRISMA guidelines [50]. In compliance with the SQUIRE 2.0 checklist [51], data were extracted using a standardized approach based on characteristics, such as publication details, study design, and outcome, among other necessary information, and utilized to acquire and authenticate the approach and results of the included studies.

**Table 1:** Inclusion and exclusion criteria.

Parameters	Inclusion criteria	Exclusion criteria
Research topic	Effects of exposure to e‐waste or e‐waste chemicals	Effects of exposure to materials other than e‐waste or e‐waste‐associated chemicals
Type of publication	Full‐text articles and textbooks	Abstracts, commentaries, and correspondences
Research method	Clear or well‐defined methods	Unclear or not‐well‐defined methods
Study language	English	Written in other languages
Reported outcomes	Mechanisms of toxicity and adverse health outcomes associated with e‐waste exposure or exposure to e‐waste chemicals Relevance of Adverse outcome pathway (AOP) networks and new approach methodologies in chemical risk assessment	Mechanisms of toxicity adverse health outcomes not associated with e‐waste exposure or exposure to e‐waste chemicals

## Result

**Fig. 2:**
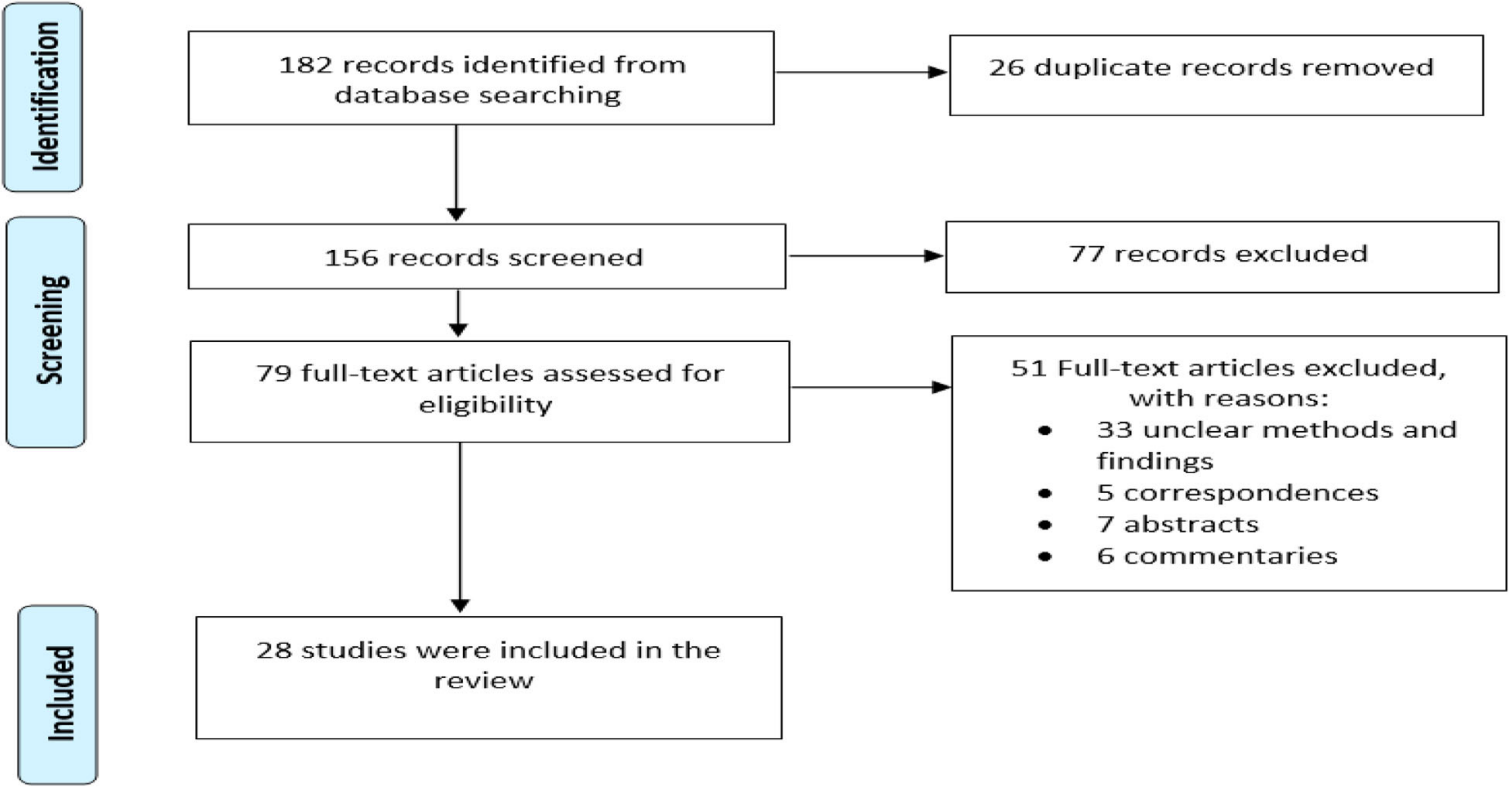
PRISMA flow diagram for the study. It is a 3‐phase flow diagram that depicts the screening procedure used to choose the number of articles for this review paper. The number of papers is recorded at various phases, and the selection process is made transparent by reporting on decision‐making at each level.

Overall, a total of 182 studies were retrieved from the extensive search, of which 26 duplicate articles were excluded. Following screening of the title and abstracts, 77 articles were not included for not matching the inclusion criteria, leaving 79 full‐text articles for further scrutiny. Suitable for inclusion in the present study were 28 full‐text articles after being examined for specific inclusion as demonstrated in the PRISMA flow diagram (Fig. 2).

## Mechanisms of toxicity

**Fig. 3:**
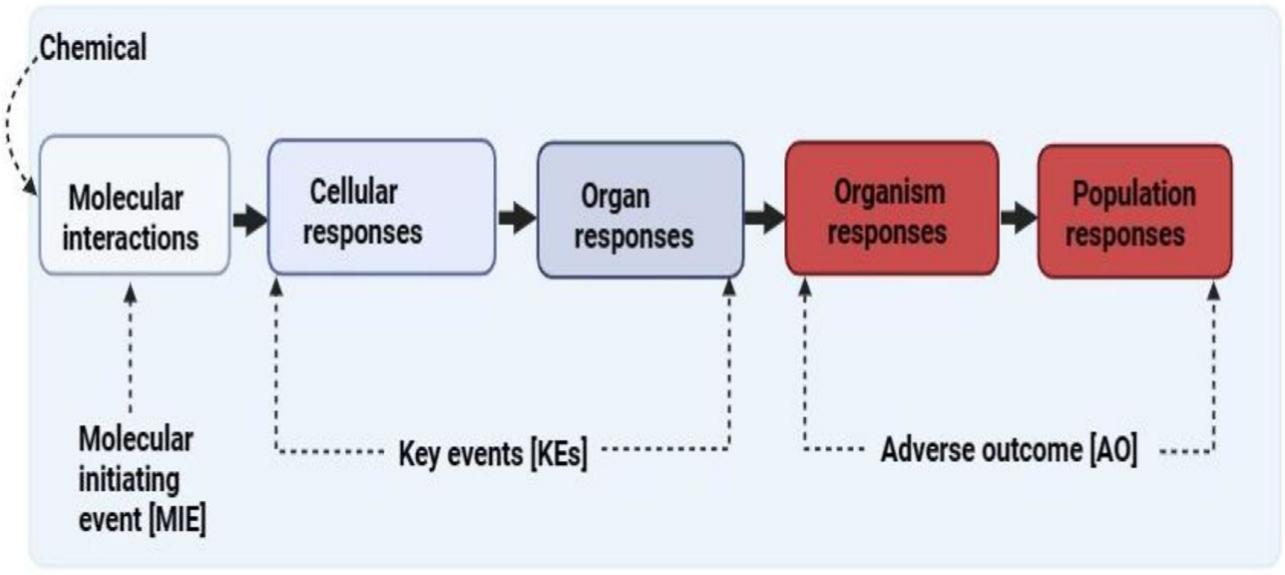
Architecture of an AOP [16]. The adverse outcome (AO) is typically the observed toxicity in the exposed organism because of a chemical acting through the molecular initiating event (MIE) and subsequently via a number of key events (KEs). As such, the toxicity profile of a chemical is better described by elucidating the associated MIE and each subsequent KE that follows within the pathway leading to the AO.

Toxicity can be defined broadly as a detrimental influence on cell formation, activity, and/or survival [16], while toxicity pathways are characterized as cellular response pathways caused by chemical exposure that are likely to have negative health consequences [52]. The idea of toxicity pathways has been broadened to encompass the framework of AOPs (Fig. 3). In the AOP strategy, the cellular‐level response pathway, which is frequently highly maintained across species, is utilized to obtain a mechanistic picture of the biological response to chemicals, allowing for extrapolation from cellular responses to anticipated population effects [53, 54, 55]. As such, an AOP connects the toxicity pathway at the cellular level to the organ‐level reaction, then the organismal response, and lastly the effect on the population [16].

**Fig. 4:**
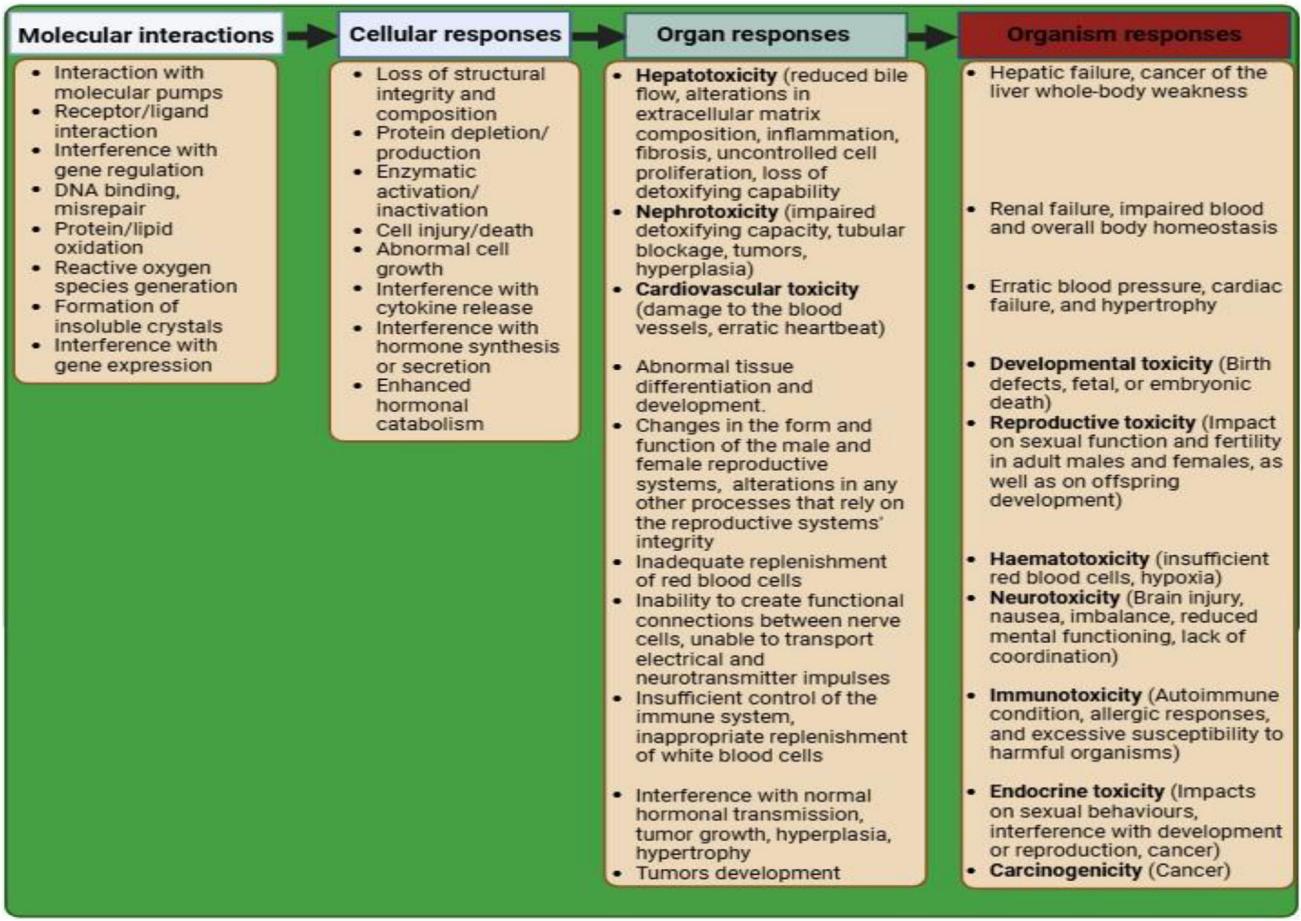
Human toxicity pathways possibly associated with e‐waste exposure. The AOP concept was utilized in a thematic manner to explore the different types of toxic responses that may be associated with e‐waste exposure, including toxicity to specific organs (e.g., liver, kidneys, and heart), organ systems (e.g., blood formation, nervous, immune, and endocrine systems), and integrated organism effects (e.g., developmental, and reproductive effects, and carcinogenicity). The identified molecular interactions and cellular responses can have impacts at the tissue, organ, organ system, and, ultimately, organism and population levels. As such, constructing a comprehensive risk assessment screening battery requires a full understanding of this concept for each possible site of toxicity. The information provided in this figure is based on classical toxicology textbooks and articles on e‐waste‐associated chemical exposures used for this study.

The significance of xenobiotic receptors is critical in establishing the molecular processes of detrimental effects associated with chemical exposure since metabolic cellular responses are mostly governed by receptors [56]. Attempts have been made to screen the interactions between environmentally relevant chemicals, including e‐waste‐associated chemicals [57, 58, 59, 60], e‐waste‐impacted soil matrix [61], and cellular receptors. The highest fold induction of Atlantic cod (*Gadus morhua*) peroxisome proliferator‐activated receptor alpha‐1 (3‐fold), peroxisome proliferator‐activated receptor alpha‐2 (28‐fold), estrogen receptor alpha (15‐fold), aryl hydrocarbon receptor 1‐alpha (28‐fold) and aryl hydrocarbon receptor 2‐alpha (39‐fold) was observed with the West African e‐waste soil‐derived extracts [61]. The varied levels of sensitivity observed in the receptor‐based bioassays could pose a diverse risk of adverse outcomes in aquatic ecosystems and finally in humans via trophic interactions since the e‐waste soil usually contain agonists for the various xenobiotic receptors [61]. As such, toxicity pathways could be triggered following e‐waste exposure due to interactions between the e‐waste chemicals and xenobiotic receptors, whereby the chemicals activate and/or increase the metabolic activity within a given cell, and this usually result in information exchange between different cells, tissues, and organs and ultimately resulting to adverse outcome in the body (Fig. 4). The obtained responses may then be observed across a population and with potential implications for population and ecosystem health. Eventually, humans exposed to e‐waste can demonstrate a wide range of tissue‐, organ‐ and organ system‐level responses, many of which are traceable to the toxicant's molecular or cellular effects, demonstrating the idea of toxicity pathways [16].

The first step is a molecular contact between the harmful chemical and receptor or other biological molecules, a process called the molecular initiating event (MIE). The interactions between the chemical and biomolecule generate a cellular response, which eventually results in measurable endpoints. In essence, when the capacity of repair and defense mechanisms has been overcome, all mechanisms eventually lead to cytotoxicity and/or cell death [16]. Although several cytotoxicity mechanisms may occur in response to anthropogenic chemicals, cell death associated with exposure to most e‐waste chemicals had occurred predominantly through apoptosis and necrosis [62, 63, 64]. Apoptosis, also known as programmed cell death, is triggered to eliminate damaged cells and is critical in the removal of pre‐cancerous cells, whereas necrosis occurs when vital cell activity is irreversibly inhibited [16]. The cytotoxicity as well as mechanisms of cell death associated with exposure to e‐waste chemicals, such as heavy metals [62], organic pollutants [63] and e‐waste soil‐derived fractions [61, 64], have been explored *in vitro*. Overall, it is plausible to conclude that the cytotoxic effects associated with e‐waste exposure may vary greatly depending on the type of chemical or mixture of chemicals encountered, the concentration, the metabolic condition of the exposed cell, and the cell's absorption mechanism for the different chemicals [62].

### Hepatotoxicity

Hepatotoxicity is modulated by multiple essential mechanisms, including toxicant absorption and concentration, biological activation and detoxification, and rejuvenation [16]. The liver is the body's primary metabolic and detoxifying organ, and chemical exposure has a negative impact on hepatic functioning, biosynthetic ability, and hepatocyte integrity [16]. Apoptosis, caspase activation, and ultrastructural abnormalities of organelles, such as the endoplasmic reticulum and mitochondria are among the changes observed in hepatocytes following e‐waste chemical exposures [65, 66]. Inflammation involving proinflammatory cytokines, tumor necrosis factor alpha (TNF‐α), mitogen‐activated protein kinase (MAPK), and extracellular‐signal‐regulated kinase (ERK) pathways have also been reported in heavy metal induced hepatotoxicity [66]. Individuals exposed to e‐waste have been reported to have dysregulated liver enzymes, including alkaline phosphatase, alanine transaminase, and gamma glutamyl‐transferase, resulting in liver damage [67, 68]. A cross‐sectional investigation had revealed that chronic e‐waste exposure was associated with a surge in albumin levels [67, 68] as well as a reduction in total bilirubin (T. Bil) and reduced glutathione (GSH) levels [68], which suggests a potential risk of liver toxicity. Molecular and cellular events, such as membrane‐initiated cytotoxic signaling pathways with *c*‐jun N‐terminal kinase (JNK) and their role in mitochondrial dysfunction, and the possible role of oxidative stress, could be an excellent index to evaluate the metabolic functions of the liver.

### Nephrotoxicity

The kidney is essential for the metabolism and excretion of flame retardants, which are a key chemical component of e‐waste [69, 70, 71, 72]. Only a few studies have investigated the nephrotoxicity of flame retardants. This is surprising given that the minimal risk thresholds for flame retardants, such as tris(1,3‐dichloroisopropyl) phosphate (TDCPP) and tris(2‐chloroethyl) phosphate (TCEP) are estimated based on alterations in kidney pathology [72]. As e‐waste exposure rises, especially in developing countries, more research into the direct and indirect pathways of nephrotoxicity following exposure to e‐waste chemicals is urgently required. Blood urea and serum creatinine [73], as well as the urine albumin Alb/creatinine ratio (UACR) [74], are commonly employed indicators of nephrotoxicity and renal dysfunction. However, they are regarded as insensitive to detecting initial renal impairment. Although different nephrotoxic biomarkers, such as kidney injury molecule‐1 (KIM‐1) and neutrophil gelatinase‐associated lipocalin (NGAL) are used to monitor the progression of acute kidney disease in clinical research and offers the possibility of identifying exposure to individual pollutants, Beta‐2‐microglobulin (β2‐MG) and *N*‐acetyl‐β‐D‐glucosaminidase (NAG) have gained widespread use in the environmental field [75]. Adult residents exposed to e‐waste had considerably greater levels of serum creatinine and urinary β2‐MG compared to the control group. These levels were also positively linked with serum PCB levels [22]. Heavy metals such as lead, cadmium, and arsenic are also known nephrotoxins that target the renal proximal tubules via distinct toxicity mechanisms [69, 70, 71].

### Cardiovascular toxicity

It is unclear how vascular toxicity affects physiological function and/or causes toxicity in other organs. Nonetheless, injury to vascular epithelial cells may produce reactive oxygen species (ROS) and subsequent oxidative damage. Toxic responses of the vascular system could also include changes in blood pressure, damage to blood vessels and damage to either epithelial cells or smooth muscle cells. On the other hand, cardiotoxicity could result primarily via altered ion channels, calcium ion homeostasis, as well as electrical excitability and action potential production [16]. E‐waste exposure raises harmful chemical levels, which leads to aberrant cardiovascular physiology measurements [11]. Cardiovascular risk in preschool children exposed to e‐waste included vascular inflammation and lipid abnormalities, which were aggravated by exposure to polyaromatic PAHs and lead [76, 77], while air pollutants resulted in increased heart rate and plasma norepinephrine in participants [78]. E‐waste burning in India contributed to severe air pollution, which was associated with an increased prevalence of cardiovascular morbidity, specifically hypertension, in exposed adult residents [33], while noise pollution from an e‐waste site in Ghana was associated with an increased heart rate in exposed adults [79]. E‐waste chemicals, such as lead, arsenic, cadmium, vinyl chloride, and fluorocarbons may cause cardiovascular disease by damaging the vascular system's endothelial barrier, stimulating the inflammatory response, activating leukocytes and platelets, initiating plaque formation, causing kidney‐related hypertension, and causing direct damage to cardiac and blood vessel tissue [16].

### Neurotoxicity

Heavy metals, such as aluminum, arsenic, lead and mercury can impact neurotransmission and impair the calcium signaling pathway, interfering with synaptic function [80]. Early childhood elevated blood lead levels can harm neurodevelopment, resulting in behavioral abnormalities, reduced cognitive function, hyperactivity, attention deficiencies, and conduct issues [81]. Flame retardants, such as PCBs and PBDEs, as well as other e‐waste associated organic pollutants are known developmental neurotoxins that can impair a variety of neuropsychological functions in children, including visual–spatial function, general cognition, motor function, memory, and attention [82, 83]. PAHs may have a negative impact on child neurodevelopment and result in IQ impairments [84, 85, 86]. Neurotoxicants primarily target the axon, the neuron, the neurotransmitter system, and the myelinating cell [16]. Neurons have a limited ability to eliminate reactive oxygen species and are especially susceptible to oxidative stress [80]. Lead exposure causes the central nervous system to produce more superoxide anion and hydrogen peroxide, which can react with proteins, DNA, and lipids to promote apoptosis [13].

### Hematotoxicity

Chronic exposure to lead, which interfere with hematopoiesis or reduce the survival of red blood cells, can cause anemia and hypoxia [87]. Red blood cell viability can be negatively impacted by oxidative damage, which interferes with hemoglobin's oxygen‐carrying capability, or by alteration of cell surface proteins, which can result in the loss of self‐antigens and subsequent death by white blood cells [16]. E‐waste workers exposed to elevated levels of lead had significantly higher diastolic blood pressure as compared to their non‐e‐waste exposed individuals [87]. Furthermore, e‐waste workers with higher blood levels exhibited a trend of reduced hemoglobin as compared to the non‐e‐waste exposed individuals with lower blood lead level. Lead and PAHs exposure were risk factors related to platelet indices among e‐waste exposed preschool children [35, 88]. Lead in erythrocytes was linked to negative alterations in red blood cell indices and hemoglobin in children from an e‐waste recycling location [89]. In addition, changes in the number and proportion of innate immune cells were connected to higher lead and cadmium levels in children exposed to e‐waste [90]. People living in an e‐waste disassembling environment reportedly exhibited a higher body load of PCBs and specific PBDE compounds, which contributed to aberrant hematological marker alterations [11]. As such, the hematological system could be affected following e‐waste exposure [87].

### Immunotoxicity

The tendency of e‐waste chemicals like lead, cadmium, and aluminum to disrupt the immune system has been a major cause of health concern for scientists and public [91]. Lead, among these chemicals, has been shown to have a considerable immunotoxic effect on people who have been exposed to e‐waste [11]. Children who had been exposed to e‐waste had significantly higher blood lead levels and lower antibody levels than the reference group [32, 92, 93]. Children that were persistently exposed to lead had lower antibody titers compared to the reference group, indicating a decreased immune response to diphtheria, tetanus, pertussis, polio, Japanese encephalitis, [37] and hepatitis B [37, 93]. Individuals with modestly elevated blood lead levels greater than 25 mg·dL^−1^ have a negative impact on the host immune system, resulting in a drop in the absolute number and percentage of CD3^+^ and CD4^+^ cells [94]. The immunotoxic effects of organophosphate flame retardants in human THP‐1‐derived macrophages [95] and dendritic cells [96] have been reported. Triphenyl phosphate (TPP) and tris(2‐butoxyethyl) phosphate (TBEP) reduced THP‐1 macrophage adhesion and phagocytosis, while tri‐n‐butyl phosphate (TNBP) and tri‐o‐cresyl phosphate (TOCP) could trigger immunostimulation by greatly increasing cell adhesion and phagocytic efficiency [95]. Functional loss of phagocytosis in human THP‐1‐derived macrophages was associated with exposure to tris(1,3‐dichloroisopropyl) phosphate (TDCPP) [95] while triphenyl phosphate (TPP) exposure induced an activated phenotype in steady state dendritic cells [96]. The proinflammatory properties of TDCPP, TPP, and TBEP has also been reported [95]. The findings could imply that immunotoxicity linked to e‐waste exposure may manifest in a variety of ways, highlighting the need for more sensitive bioassay approaches to offer additional evidence.

### Endocrine toxicity

Following e‐waste exposure, chemicals linked with e‐waste could bind hormone receptors, altering secretion, distribution, metabolism, and interfering with normal hormone levels in the human body, disrupting the function of the endocrine system. Infertility, obesity, diabetes, heart disease, neurological disorders, and cancer have been linked to endocrine disruption [97]. The reported effects on thyroid‐stimulating hormone (TSH) levels following e‐waste exposure vary, with some research reporting greater levels of thyroid‐stimulating hormone [98, 99], and others revealing reduced thyroid‐stimulating hormone levels [102, 103, 104, 105] in exposed populations. Tetraiodothyronine (T4) values in the exposed population were not greater than those in the reference population, and serum PBDEs had a relationship that was inverse with T4 [98, 100, 101, 102]. Serum PCB levels were also found to be inversely linked with thyroid‐stimulating hormone [44], free triiodothyronine [22], and free thyroxine [22, 42] in exposed individuals. There are also reports of no association between TSH and PCBs [104], or PBDEs [22]. Thus, e‐waste exposure may result in improper endocrine interactions and/or disrupt the intricate system of hormonal feedback loops. A significant portion of the endocrine system's function relies on sensitive feedback loops, and contaminants that impact or mimic hormones usually affect many endocrine glands [16].

### Developmental toxicity

Increased body levels of some e‐waste chemicals, such as lead, PBDEs, PAHs, and cadmium, have been reported to be associated with altered fetal growth during early life [11]. Exposure to e‐waste during pregnancy has been linked to lower birthweight, body mass index, and head circumference [34, 106] as well as decreased child growth and development [25, 26, 105]. Additionally, a 10 ng·g^−1^ increase in placental cadmium concentration has been linked to a 205 g weight loss and a 0.44 cm decrease in body length [24]. Elevated blood lead levels were reported be associated with poorer neurodevelopmental outcomes [11]. Children with greater blood levels of e‐waste chemicals, such as lead and cadmium, were shown to have behavioral abnormalities [28]. In another study, children with high blood lead (≥10 μg·dL^−1^) had 2–4 times the risks of attention‐deficit hyperactivity disorder than those with minimal lead exposure [107]. The means by which a toxicant causes dysmorphogenesis, mortality, growth retardation, and functional changes can simply be referred to as the mechanism of developmental toxicity [16].

### Reproductive toxicity

A range of e‐waste‐associated chemicals have been shown to imitate or block androgens, estrogens, and progestogens, causing reproductive problems. Toxicants such as PCBs, brominated flame retardants, dioxins, hexachlorobenzene, and heavy metals have been linked to reproductive abnormalities, although their exact mechanism is largely unknown [16]. However, most toxicants that impact spermatogenesis have a direct effect on the testis or spermatogenesis itself by interfering with or destroying Sertoli cells or interfering with energy production in sperm cells [16]. The estrogen‐like effect of bisphenol A (BPA), an e‐waste associated chemical, may result in reproductive toxicity [108]. BPA may cause female premature puberty and reproductive system cancers, which can impair fertility, as well as lower sperm quality and quantity in men [108]. Male reproductive disease was identified among e‐waste‐exposed outpatients at Guiyu Hospital in China, with greater incidence of azoospermia, epididymitis, and asthenozoospermia than at the reference hospital [109]. The reproductive system's goal is to produce high‐quality gametes that can fertilize and produce viable offspring, which can then effectively reproduce. The fact that some e‐waste chemicals can adversely affect the high‐quality gametes that can fertilize and produce viable offspring is not a new notion, but this requires mechanistic investigations into the toxicity pathways involved, especially during combined exposures [16, 109].

### Genotoxicity

E‐waste chemicals, such as chromium, cadmium, arsenic, lead, and mercury [110, 111, 112, 113, 114, 115, 116, 117, 118, 119], and flame retardants, like PBDEs [120, 121], can induce genetic damage at either nuclear, chromosomal, and or molecular levels via multiple pathways. These pathways include the creation of micronuclei, chromosomal aberrations, sister chromatid exchanges, point mutations, DNA adducts, protein adducts, DNA strand breaks, oncogene activation, mutations/oncoproteins, and DNA repair and other anomalies and molecular‐level DNA damage [122]. A few studies have reported increased risks of genotoxicity among e‐waste exposed populations than in control populations using biomarkers in different biological matrices, including buccal cells, blood, umbilical cord blood, placenta, urine, and semen [123, 124, 125, 126, 127, 128, 129, 130]. The incidence of DNA damage in newborns from an e‐waste‐exposed area in China was greater compared to the reference group, and there was a positive association between blood chromium and the observed DNA damage [131]. Environmental exposure to harmful e‐waste chemicals during pregnancy may also increase the chance of shortening placental telomere length [132]. An e‐waste‐exposed group in China also had greater total chromosome aberration and micronuclear incidences than the reference group, and women had greater chromosome aberration and micronucleus incidence than men [133]. Therefore, elevated amounts of toxic e‐waste chemicals in the body, both in individual and combined forms, may cause cell and DNA damage.

### Carcinogenicity

E‐waste associated chemical such as PAHs that interact physically with DNA to alter or damage its structure are referred to as genotoxic carcinogen while those that impact DNA expression through protein phosphorylation, DNA methylation and receptor‐mediated effects, without directly affecting DNA structure are referred to as epigenetic carcinogens. Both genotoxic and epigenetic carcinogens can eventually lead to cancer. Some non‐carcinogenic metal ions found in e‐waste, such as copper and iron, can cause oxidative stress and subsequently induce DNA damage, lipid peroxidation, protein modification, and other possible effects that can lead to diseases including cancer [134]. Overall, carcinogenesis develops through various toxicity pathways, establishing the introduction of a mutation in the DNA sequence (initiation), followed by the selective expansion of initiated cells (promotion), and finally, the conversion of unstable promoted cells into stable malignant tumors [16]. E‐waste chemicals, such as arsenic, cadmium, chromium, nickel and polyaromatic hydrocarbons are known carcinogens [135, 136], and can lead to lung, skin, or bladder cancer [135]. A few studies have reported that elevated concentrations of harmful chemicals from e‐waste recycling sites could result in increased cancer risks in exposed individuals [137, 138, 139, 140, 141]. A recent study reported that children living near the formal e‐waste dismantling site still suffered a carcinogenic health risk from metals in the soil [142]. Many carcinogens are not fundamentally carcinogenic [16]. Rather, they require metabolic activation to become carcinogenic. This activation may potentially have tissue‐specific consequences, as various tissues have varying degrees of enzyme expression [16].

## Safety testing

Safety testing of chemicals has changed dramatically in recent years, prompted by concerns about ethical issues, financial requirements, laborious methods, and the scientific limits of traditional animal‐based assays [17, 143]. Also, concerns have been expressed about the dependability, sustainability, reproducibility, and translational applicability of animal‐derived safety information to humans [143, 144, 145, 146, 147]. The use of animals for chemical risk assessment just takes too long compared to *in vitro* testing and could be too expensive for the sheer number of chemicals on the market now and those expected to emerge in the future. Furthermore, the current traditional animal testing approach could fall short in the assessment of probable harmful effects from multiple chemical exposures, as evaluating huge numbers of possibly harmful chemical combinations in experimental animals is clearly not practicable [17]. A thorough evaluation of potential deleterious effects from combined exposure to several chemicals should ideally be based on complete information about the toxicological mechanism, such as the molecular target(s) of all chemicals involved in the combined exposure [148]. However, traditional animal testing rarely provides such mechanistic insights, therefore adding ambiguity to the approach. These concerns necessitate the need for an essential paradigm shift in chemical risk assessment [17].

The present review paper identified possible adverse health outcomes associated with e‐waste exposure. However, the impacts of prolonged exposure to complex e‐waste chemical combinations have yet to be thoroughly investigated and comprehended [123]. Exposure assessment is one of the four fundamental elements in the chemical risk assessment framework and an essential component of any quantitative risk assessment [149]. It is used to quantify or assess the magnitude, frequency, and duration of chemical exposure, as well as the size and composition of the exposed population [150]. Thus, this could explain the sources, routes, pathways, and ambiguity associated with a chemical risk assessment [151, 152, 153]; describe exposure to chemicals in real‐world situations at various developmental stages; and offer data to explain health outcomes in various populations [150]. Given that exposure assessment frequently informs risk assessment [150], an effective e‐waste exposure assessment study would necessitate a thorough application of the risk assessment process. This would include identifying adverse effects that might occur following e‐waste exposure (hazard identification); estimating the toxicity of exposure to real‐life e‐waste chemical mixtures by evaluating the quantitative relationship between exposure and response, usually obtained through animal toxicity tests (dose–response assessment); determining the magnitude, frequency, duration, and rate of exposure (exposure assessment); and then e‐waste risk characterizations. Exposure assessment is typically compared to dose–response assessment; thus, the two processes use identical measurements wherever possible or describe the uncertainty associated with using alternative measures [150]. As a result, an e‐waste exposure assessment can inform risk screening, prioritizing, standardizing, permitting, enforcement, remedial decisions, and the evaluation of policies.

**Fig. 5:**
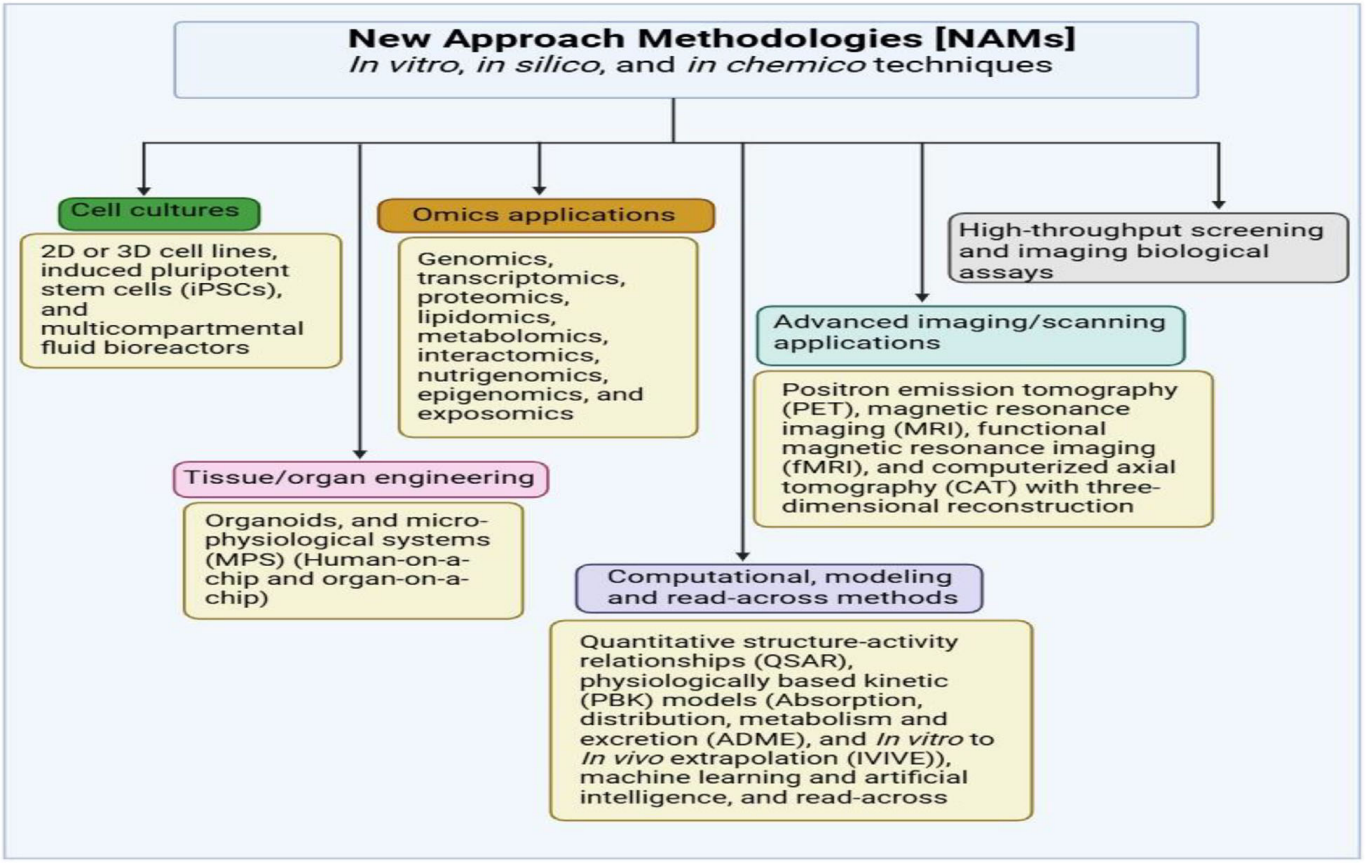
Overview of NAMs [17]. In general, NAMs encompass *in vitro*, *in silico*, and *in chemico* methods. *In vitro* data could be obtained using human‐derived cells or organoids, which can be utilized to directly identify cellular targets and chemical‐induced molecular pathways that may lead to adverse circumstances. *In silico* approaches are computational tools aimed at simulating endpoints, such as metabolic processes or at predicting effects based on chemical structural properties and linkage with established data. *In chemico* testing refers to the application of abiotic chemical reactivity technologies. Omics technologies provide insight into a wide range of complex biological responses induced by chemical disturbances. Therefore, compared to traditional toxicological studies, which produce knowledge on apical unfavorable outcomes in experimental animals, NAMs provide evidence as to why an adverse outcome is likely to take place, allowing for systems toxicology.

To meet the risk assessment and management needs for e‐waste exposure, traditional risk assessment approaches based on animal studies alone may fall behind modern risk evaluation standards [143, 144, 145, 146, 147]. NAMs are emerging as alternative strategies that combine *in silico*, *in chemico*, and *in vitro* methods to deliver mechanism‐based insights while improving ethics and human‐relevant safety information [143, 144, 145, 146, 147, 148, 154, 155, 156, 157, 158]. The establishment of NAMs and their application in chemical risk assessment adheres to the 3R principle, advocating refinement, reduction, and replacement of animal testing [159], and encompasses a variety of techniques, such as high‐throughput screening bioassays, quantitative structure–activity relationship predictions, cell culture models, omics technologies, organoids, micro‐physiological systems, and artificial intelligence [144, 145, 146] (Fig. 5). NAMs could improve understanding of the mechanisms underlying undesirable effects and determine dosages or concentrations at which impacts are unlikely to occur from a human or ecological toxicity standpoint [160]. The deployment of NAMs into the chemical risk assessment paradigm offers the potential to expedite data creation and interpretation [17] and can further indicate where additional testing may be required in an integrated or hierarchical testing plan [161]. As such, NAMs is positioned to deliver data that will give hazard, exposure, and risk information for prioritizing e‐waste chemicals for future action, and it can use a combination of information from several independent sources to provide sufficient evidence in chemical risk assessments [162].

NAMs do not only function as non‐animal alternatives. They can also be integrated in *in vivo* experiments and clinical examinations to help construct or enhance AOPs [162, 163]. When NAMs are integrated into animal test designs, they are further modified to offer mechanistic insights and dose–response information, and when they are human‐based, they can determine the human significance of a response. However, in both circumstances, they eventually improve predictive power [17]. Nonetheless, the whole idea is a critical step towards reducing the total number of animals used in research, product development, and chemical risk assessment while retaining scientific integrity and ensuring that regulatory decisions continue to protect humans and the environment from dangerous chemicals [160]. Therefore, the integration of NAMs could promote data transparency and adaptability between laboratories, broadening their usefulness beyond research [156, 161], and hold the potential to shape the future of safety testing for complex e‐waste chemical mixtures. Currently, complex mixes are increasingly being considered for chemical regulatory risk assessments [164]. However, the safety assessment of complex chemical mixtures is difficult given their non‐definitive compositional form, unpredictability, and possible synergistic and antagonistic toxicity characteristics. Furthermore, aspects like substance solubility, biomolecule accessibility, and test model utilization are expected to be carefully considered while conducting safety testing of chemicals using NAMs and may necessitate additional technical abilities. However, certain physicochemical features can also impede animal testing [17]. As a result, a thorough comparison of the safety testing of such chemicals *in vivo* and in NAMs is highly advised to determine respective limitations and adequately inform decision‐making. Notwithstanding, several NAMs have made both developmental and regulatory progress and may be utilized in mixture risk assessments if guidelines for their usage to explore complex chemical mixtures are developed [161]. In addition to providing more rapid and effective toxicity testing methods, NAMs can profoundly revolutionize today's regulatory activities by enabling better human‐relevant decision‐making in hazard and exposure assessment. Although there is a general agreement among scientific and animal welfare groups, policymakers, and an important segment of the regulatory community that the use of NAMs in risk assessment represents a significant opportunity to advance human health protection, several barriers prevent a wider use of NAMs in today's regulatory risk assessment [160].

Notwithstanding the huge and constant effort dedicated to the creation of NAMs over the last decade, their application in regulatory toxicology has been delayed [17]. Regulatory agencies often rely on firmly established, standardized, and approved toxicological methodologies to prescribe internationally recognized risk assessment procedures [160]. As such, validating and regulating NAMs is crucial for increasing their use and credibility. One of the primary reasons for the slow adoption of NAMs in the regulatory system is that some standard regulatory guidelines still recognize the existing traditional animal testing method as the acceptable approach for drawing scientific conclusions on specific safety‐related endpoints. Although NAM‐based battery approaches could also be applied to draw such scientific conclusions in a more sufficient manner than the ones provided via the traditional animal testing method, those regulations would need to be revised to accommodate the criteria for applying NAM‐based battery approaches. However, this process could be a time‐consuming process [17]. Remarkably, REACH (Registration, Evaluation, Authorization, and Restriction of Chemicals), a European Union regulation adopted to improve the protection of human health and the environment from the risks posed by chemicals, also promotes alternative methods for substance safety assessment to reduce the number of animal tests [165]. The scientific intricacy behind NAMs is rapidly expanding and are frequently multi‐disciplinary in nature, necessitating skills in a variety of domains ranging from cell biology to bioinformatics; as such, there are widespread doubt about the sustainability and familiarity with the technologies of NAMs since risk assessors are historically trained to interpret results obtained from experimental animal studies [17]. However, this would necessitate intensive training as well as the continual utilization of case studies by international and regulatory authorities to promote interactions between regulatory and academic scientists [17]. The difficulties in addressing repeated dose toxicity, especially chronic and systemic toxicities, as well as reluctance from relevant stakeholders, are also important barriers to the wider use of NAMs. Furthermore, concerns about predictability, consistency of measurements, and quantification must be tackled, and regulatory and legislative structures must be tailored to NAMs [17, 160, 161, 162, 163].

## Recommendations

To realistically assess the potential risk to human health posed by exposure to e‐waste, the most relevant information related to possible adverse effects and exposure in humans should be used. The toxic chemicals found in e‐waste or formed and released during crude e‐waste recycling can have a significant adverse impact on the health of people living in exposed areas [123]. More studies are needed to better understand the possible synergistic and inhibitory effects associated with exposure to complex e‐waste chemical mixtures, the impacts of long‐term exposure, and the results of low‐dose e‐waste exposure. This gives information about the individuals who have been exposed, assisting in determining the most effective measures to limit exposure and thus risk, as well as identifying the sources, routes, and pathways of exposure [166]. The knowledge gap becomes even larger in areas where other anthropogenic activities capable of releasing other kinds of harmful chemicals into the environment exist together with e‐waste recycling activities [61, 64]. However, the potential outputs of an exposure assessment would include a list of detected chemicals and chemical groups for use in risk analysis, as well as characterization and a conceptual model for risk. These outputs will aid in addressing location‐specific information gaps in e‐waste exposure assessment while also helping to mitigate the extraordinarily high costs associated with environmental exposure to e‐waste chemicals. Children are more vulnerable to e‐waste exposure and environmental exposures in general than adults because they have more exposure routes, a greater basal metabolic rate, and developing systems that may be unable to process and eliminate certain harmful chemicals effectively [167]. Children also have greater time than adults to acquire disorders that can be caused by hazardous chemicals in childhood and progress through a variety of phases and periods [168].

**Fig. 6:**
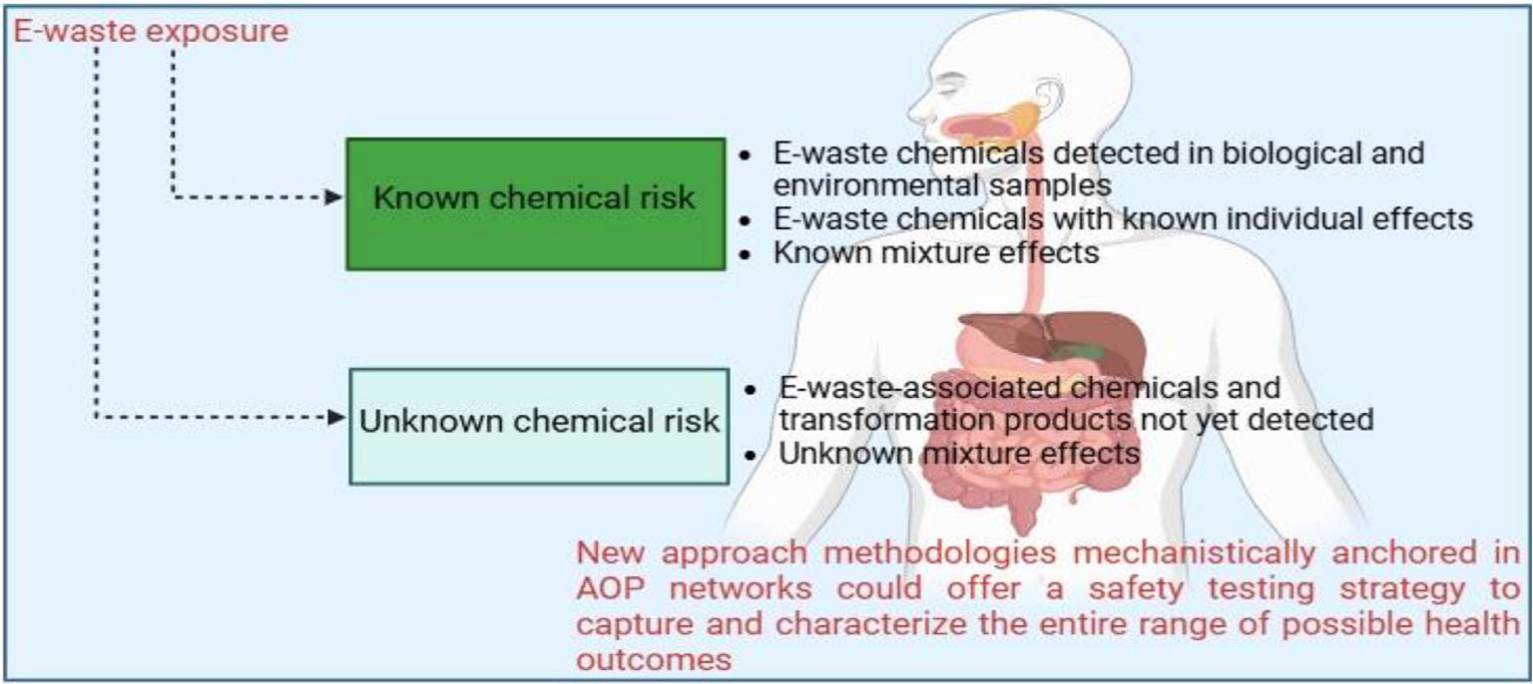
Exploring e‐waste exposure. The number of already identified chemical pollutants in e‐waste sites may be few compared to the actual available numbers. Many other chemicals, such as transformation products and both detectable and undetectable chemicals, including known and unknown ones, may be present and act in mixtures. NAMs, mechanistically anchored in AOP networks, can capture and characterize the whole spectrum of potential negative health consequences.

Humans exposed to e‐waste can demonstrate a wide range of tissue‐, organ‐, and organ system‐level responses, many of which can be traced back to the toxicant's molecular or cellular effects. Accordingly, monitoring those molecular or cellular events is necessary to enable a better understanding of KEs in the metabolic pathways that mediate known adverse health outcomes in e‐waste exposure scenarios and to enable adequate identification of the individual or groups of substances causing any adverse outcome for regulatory grouping. This is crucial to establishing accurate scientific knowledge on mixed e‐waste chemical exposures in shorter time frames and with greater efficacy, as well as supporting the need for safe management of hazardous chemicals. A practical strategy in this regard would be the utilization of NAMs, mechanistically anchored in AOP networks, which capture current understanding regarding the molecular basis of toxicity, to explore e‐waste exposure and characterize the entire range of possible health outcomes (Fig. 6). While facilitating regulatory adoption of NAMs, focusing standardization and validation is critical for overcoming the hurdles associated with traditional animal testing regimes. The internationally acceptable standard guidance for the use of NAMs, as well as critical considerations when assessing the safety of chemical combinations, must be established, and debated as needed [160]. These developments have the potential to improve the safety of e‐waste workers and susceptible population, as well as ensure the long‐term viability of chemical risk evaluations. Further progress to harmonize, validate, and regulate NAMs will allow their broad adoption in academia and industry. The application of digital twins (DTs), an innovative representation constructed to accurately portray an actual physical system in real‐time, evaluate its behavior, and offer predictive information using cutting‐edge simulation, machine learning, and logical thinking to aid decision‐making, has spread across many industries and fields, with an increasing popularity in the healthcare sector [169]. The digital twin concept for health also has the potential to transform chemical safety assessment, disease treatment and prevention, and health maintenance and consequently enhance human life [169]. DTs can accelerate this process through the identification of molecular targets with a higher likelihood of success [169]. As such, a virtual model of the human biological system might be furnished with numerous sensors relevant to critical areas of functionality. These sensors generate data on various aspects of a human biological system's performance, such as toxicokinetic processes, including toxicity pathways, and more. After receiving the necessary data, the digital model can be used to run various simulations, investigate chemical exposure scenarios, diagnose performance issues, and propose potential improvements. The goal is to gain valuable knowledge that may be applied to enhance the original or actual human biological system [169, 170]. This approach could be used in e‐waste risk assessment and chemical safety evaluation in general to offer comprehensive information regarding chemical absorption, distribution, metabolism and elimination processes that occur throughout an organism [169]. Several computer‐aided technologies have been combined with machine learning techniques to improve high‐throughput evaluation of drug absorption, distribution, metabolism, and excretion, as well as toxicity features for drug development [170]. The rapid development of big data, as well as continuous advancements in data science and artificial intelligence, have the capacity to greatly speed up chemical safety assessment by enabling scientific expertise and providing essential data, as well as strong technological advances to mechanistically demystify complex toxicological processes and provide realistic input–output predictions for biological interactions [169, 170, 171]. Utilizing DTs to simulate numerous probable molecular interactions and KEs linked with exposure to complex e‐waste chemical mixes could provide a complete understanding of the metabolic processes that occur in real‐life e‐waste exposure scenarios, benefiting both researchers and humanity. Reacting mixtures can have a severe and unpredictable negative impact on human health. Therefore, in multiple exposure scenarios, it is critical to identify the potential negative consequences for all candidates involved [17]. This could also be accomplished through predictive toxicology, which aims to profile the potential negative effects of chemical substances before they occur, both through traditional *in vivo* experimental approaches and, increasingly, through the advancement of *in vitro* and computational techniques that can minimize reliance on animal testing [16, 17, 172].

## Conclusions

Given the complex mixture of chemical pollutants associated with e‐waste recycling and their potential to cause harm acutely or over long periods, there is a need to deploy sensitive safety testing strategies that would advance scientific understanding of toxicological mechanisms associated with real‐life e‐waste chemical exposures. Since the chemical composition of e‐waste varies regularly due to technological advancements, continuous monitoring, and a clear understanding of the state of pollution and the kinds of pollutants in e‐waste sites would no doubt be key resources that would guide toxicologists and regulators in developing new safety testing strategies and new standards, as well as updating existing ones to levels where they become effective tools for protecting biota. This is necessary to raise awareness about the severity of the situation and improve the methods used for assessing and characterizing e‐waste‐related chemical pollution in affected ecosystems. Multidisciplinary and multi‐regional research on the negative health impacts of e‐waste is encouraged to reach reliable scientific conclusions. Health promotion initiatives are required among e‐waste recyclers to reduce the possibility of negative outcomes. As additional recommendations and specific methodologies with global approval become available, regulatory filings utilizing NAMs to study real‐life e‐waste chemical exposures and toxicity testing in general should continue to increase. The regulatory implementation of NAMs in chemical risk assessment will reinforce and improve the long‐term viability of chemical safety assessments while also protecting consumer health.

## Conflict of interest

The authors declare no conflict of interest.

## Author contributions

Conceptualization: CE and MV; Methodology: CE and MV; Resources: CE and MV; Writing—original draft preparation: CE and MV; and Writing—review and editing: CE and MV. CE and MV have also read and agreed to the published version of the manuscript.
